# The Red Cross and St. John's Organisation

**Published:** 1915-10-23

**Authors:** 


### THE RED CROSS AND ST. JOHN'S ORGANISATION.

### The Joint Societies' Varied Field.

Since October 21 is the anniversary of the union of the British Red Cross Society and the Order of the Hospital of St. John of Jerusalem in England, it becomes interesting to measure the extent of their joint activities during the past year. A catalogue of the organisations for which the Joint Societies are responsible would be a lengthy document; and the field of work is as varied as its branches are numerous. At home and abroad, on shore and afloat, for ourselves and our Allies, in Europe and in Asia, on every front, and behind the lines, services for the relief of the wounded and sick are busily at work under the direction of the Joint Societies. This is enough to prove Lord Wantage to have been right in his famous dictum that voluntary organisations are alone capable of giving those auxiliary forms of relief which humanity and patriotism demand for our wounded—aids which are a necessary addition to the official medical service provided by the State.

In co-operation with the Admiralty, War Office, and the medical services, a very varied collection of activities is carried on. The need for them—indeed, official dependence on them—was well proved when the German dash into France last November was followed by a daily influx of thousands of wounded into Calais and Boulogne. At that moment, when the fate of England and France hung in the balance, the Joint Societies were overwhelmed with demands for every class of medical stores. It was not a question then of supplementing official supplies: it was a question of producing medical stores in quantities which surpassed all previous calculation. The elasticity of voluntary effort, relying on the boundless generosity of the public in a cause which touched them to the heart, enabled the crisis to be passed successfully: nearly 60,000 bales have been dispatched to different centres, and £350,000 (made up of money spent and the value of gifts received by the Societies) has been provided for the supply of necessary goods.

In the Special Number of The Hospital devoted to transport questions we gave accounts of the hospital trains, ships, motor ambulances and launches which have played such a remarkable part in the quick transport of wounded from the Front to base hospitals in France and England. This lightning dispatch of wounded, the latest development of the war in this field is, indeed, what Red Cross work has come to mean to the general public. There are now 625 ambulances running, with a *personnel* of 1,200. To see the work at a glance we may recall that on one day alone 7,106 wounded were brought into Boulogne.

### Institutional Organisations.

The prominent part played by motor transport must not obscure the vast work of the Joint Societies' hospitals in France, without which the number of beds available in the military hospitals would have been often entirely insufficient. The hospitals for the British, eight in number, are, in their order, at Le Touquet, Rouen, Abbeville, Wimereux (two), Etaples (two), and Paris Plage. Besides these are four hospitals for the Allies, and numerous rest stations at railway junctions and other centres. In addition to these, all the voluntary hospitals offered by private donors to the French and the Belgians have to be approved by the joint committee of the two Societies in accordance with the wishes of the French Government. Of these privately given hospitals there are over twenty. The institutional problems of the war, however, are very varied, as the hospital specially equipped at Brockenhurst Park for wounded Indians reminds us. Retired officers of the Indian Medical Service form the staff; all the sisters speak Hindustani; the respective customs of the Mohammedans and Hindus are respected. The special stores dispatched to hospitals with wounded Indians among their patients include sacred books, Sikh symbols, wooden combs for those (Sikhs) who do not cut their hair, funeral urns, and gramophones with Indian tunes. Then the King George Hospital in Stamford Street, once the new building of the Stationery Office, with its 1,650 beds, is the crowning edifice of these institutions, though at the other extreme the series of wooden huts, each for twenty patients, known as the Red Cross Hospital, Netley, must not be forgotten. With the hospitals equipped at Malta, Alexandria, and Cairo for the soldiers wounded at the Dardanelles we cannot here deal.

### Needs and Helpers.

Extensive subsidiary organisations are busy in connection with this hospital system, of which the chief is perhaps the bureau for tracing wounded and missing, and for supplying information as to the position of the graves in France. Photographs of these are sent free of cost to relatives. Supplies of food are sent to prisoners in Germany; river trips up the Thames have been arranged during the summer for convalescents; the 4,000 buildings offered privately for auxiliary hospitals have been inspected and reported on, of which 105 have been formally accepted by the War Office, and 955, with a capacity of 32,500 beds, are now at work. The special needs of the permanently injured have led to the establishment of the Hostel for Blinded Soldiers and Sailors at St. Dunstan's, Regent's Park, with its eighty patients (gone once more, alas! to school). The "Star and Garter," Richmond, is being rebuilt as a permanent home for the paralysed and disabled; and those who have lost a limb have an institution provided for them at Roehampton. Of the medical and nursing staffs, and the hospital orderlies, there is no space to speak; of the hospital ships and motor launches now plying in the Persian Gulf and the Ægean Sea, with all the transport work involved in these areas, we can say no more than appeared in The Hospital's Special Transport Number of April last.

